# Metagenomic and Metabolomic Perspectives on the Drought Tolerance of Broomcorn Millet (*Panicum miliaceum* L.)

**DOI:** 10.3390/microorganisms13071593

**Published:** 2025-07-06

**Authors:** Yuhan Liu, Jiangling Ren, Binhong Yu, Sichen Liu, Xiaoning Cao

**Affiliations:** 1 Center for Agricultural Genetic Resources Research, Shanxi Agricultural University, Taiyuan 030031, China; liuyuhan202305@163.com (Y.L.); renjiangling0@163.com (J.R.); yubinhong1223@163.com (B.Y.); lsch209@163.com (S.L.); 2 College of Agriculture, Shanxi Agricultural University, Jinzhong 030801, China

**Keywords:** broomcorn millet, drought stress, rhizosphere microorganisms, metagenomics, metabolomics

## Abstract

Drought stress is an important abiotic stress factor restricting crop production. Broomcorn millet (*Panicum miliaceum* L.) has become an ideal material for analyzing the stress adaptation mechanisms of crops due to its strong stress resistance. However, the functional characteristics of its rhizosphere microorganisms in response to drought remain unclear. In this study, metagenomics and metabolomics techniques were employed to systematically analyze the compositional characteristics of the microbial community, functional properties, and changes in metabolites in the rhizosphere soil of broomcorn millet under drought stress. On this basis, an analysis was conducted in combination with the differences in functional pathways. The results showed that the drought treatment during the flowering stage significantly altered the species composition of the rhizosphere microorganisms of broomcorn millet. Among them, the relative abundances of beneficial microorganisms such as *Nitrosospira*, *Coniochaeta*, *Diversispora*, *Gigaspora*, *Glomus*, and *Rhizophagus* increased significantly. Drought stress significantly affects the metabolic pathways of rhizosphere microorganisms. The relative abundances of genes associated with prokaryotes, glycolysis/gluconeogenesis, and other metabolic process (e.g., ribosome biosynthesis, amino sugar and nucleotide sugar metabolism, and fructose and mannose metabolism) increased significantly. Additionally, the expression levels of functional genes involved in the phosphorus cycle were markedly upregulated. Drought stress also significantly alters the content of specific rhizosphere soil metabolites (e.g., trehalose, proline). Under drought conditions, broomcorn millet may stabilize the rhizosphere microbial community by inducing its restructuring and recruiting beneficial fungal groups. These community-level changes can enhance element cycling efficiency, optimize symbiotic interactions between broomcorn millet and rhizosphere microorganisms, and ultimately improve the crop’s drought adaptability. Furthermore, the soil metabolome (e.g., trehalose and proline) functions as a pivotal interfacial mediator, orchestrating the interaction network between broomcorn millet and rhizosphere microorganisms, thereby enhancing plant stress tolerance. This study sheds new light on the functional traits of rhizosphere microbiota under drought stress and their mechanistic interactions with host plants.

## 1. Introduction

A drought represents a prevalent environmental stress in agricultural systems, inflicting greater yield losses than the cumulative impact of all biotic stressors combined [1]. In response to soil moisture fluctuations, plants employ multifaceted adaptive strategies to mitigate drought effects [2,3]. These include accumulating osmoprotectants [4], thickening cuticular layers [5], and enhancing photosynthetic apparatus resilience to dehydration [6]. Drought stress enhances ABA biosynthesis in plants [7], regulates guard cell movement, and modulates gene transcription during different plant developmental stages [8]. It also suppresses CTK signal transduction, inhibits cell elongation, and reduces root growth [9,10]. Concurrently, the composition and concentration of plant root exudates are altered [11]. As a major source of soil organic carbon, these exudates reshape the soil microenvironment and modify the microbial community structure [12]. The concentrations of osmolytes (e.g., proline, reducing sugars, and organic acids) increase [13], thereby enhancing plant osmotic stress tolerance and modulating rhizosphere microbial growth [14].

In the rhizosphere, microorganisms form symbiotic relationships with plants, establishing a complex microbial community. This community mediates plant–soil interactions [15,16] and indirectly regulates plant physiological functions [17]. For instance, the rhizosphere microbial community facilitates plant nutrient uptake [18], bolsters disease resistance and stress tolerance [19,20], accelerates soil organic matter decomposition, and enhances soil fertility [21]. Furthermore, its composition dynamically adapts to plant developmental stages [22], genotypes, and environmental conditions [23], thereby optimizing plant growth and development. Metagenomic and metabolomic approaches have been widely applied to elucidate the interaction mechanisms between plants and rhizosphere microorganisms. Li et al. demonstrated that applying γ-PGA under drought conditions enhances the structural stability of rhizosphere soil microbial communities in maize. This treatment increases the abundance of bacteria involved in C, N, and P cycling, improves soil texture and nutrient availability, and optimizes osmotic regulation. Consequently, it promotes maize water and nutrient uptake, ultimately enhancing drought resistance [24]. Nitrogen deposition and its synergistic interaction with drought stress enhance nitrogen fixation and phosphorus solubilization capacities in rhizosphere soil microorganisms. These effects are associated with altered expression of key functional genes (particularly those involved in the carbon cycle), ultimately improving nutrient acquisition and utilization by *Fargesia* spp. [25]. The application of biofertilizers increases the levels of amino acids and phenolic compounds in cotton roots and the surrounding soil. These metabolites reduce cadmium uptake and improve cotton’s heavy metal resistance [26].

Broomcorn millet (*Panicum miliaceum* L.) is primarily cultivated in semi-arid regions of Asia (including East Asia, West Asia, and South Asia) and Europe [27]. As a key minor cereal crop in the Loess Plateau region, broomcorn millet demonstrates greater drought tolerance than other crops [28]. As an ideal crop for studying stress resistance mechanisms [29], broomcorn millet has been the focus of research on rhizosphere microbial community composition [30]. Among these studies, Tian et al. demonstrated that mean annual temperature and soil pH are the primary drivers regulating the rhizosphere microbial community, including key species and modularity, in broomcorn millet [31]. Additionally, soil nutrients [32], planting and cultivation methods [33,34], and growth stages [35] significantly influence the diversity and community structure of rhizosphere microorganisms. The flowering stage represents a critical period for broomcorn millet growth and yield formation [34,36], during which rhizosphere microbial activity peaks. Investigating this phenological phase provides valuable insights into potential interactions between rhizosphere microbiota and broomcorn millet productivity. Current research on broomcorn millet rhizosphere microorganisms under drought stress during flowering has primarily investigated microbial diversity shifts and predicted metabolic functions [36,37]. However, the dynamic changes in rhizospheric chemical composition and associated metabolites remain poorly understood. Using integrated metagenomic and metabolomic approaches, we investigated drought-induced alterations in rhizospheric soil microbial communities and metabolite profiles during broomcorn millet’s flowering stage. Our analysis revealed significant structural changes in microbial populations and their interactions with broomcorn millet root exudates, elucidating key microbial–metabolite relationships under drought conditions. Metagenomic analysis revealed significant enrichment of drought-tolerant microorganisms (e.g., *Coniochaeta* spp.) and functional genes associated with stress adaptation, including acetic acid metabolism (*aclB*) and phosphorus solubilization (*phoD*). Parallel metabolomic profiling quantified key microorganism-derived osmoprotectants (trehalose, proline) and critical pathway intermediates, demonstrating a coordinated rhizosphere response to drought stress. We hypothesize that drought-stressed rhizospheric fungi and bacteria synergistically enhance osmotic regulation and drought tolerance by differentially expressing key functional genes (*aclB*, *phoD*), activating glycolysis/gluconeogenesis and fructose/mannose metabolism pathways, and modulating rhizospheric metabolite composition (e.g., trehalose, proline), thereby collectively regulating host physiological responses. These findings advance our understanding of broomcorn millet–rhizosphere microorganism interactions under drought stress, providing critical theoretical insights for elucidating broomcorn millet’s drought tolerance mechanisms and supporting sustainable organic dryland farming practices.

## 2. Materials and Methods

### 2.1. Test Materials, Drought Stress Treatment, and Sample Collection

The experiment was conducted at the Experimental Base of the Agricultural Gene Resources Research Center, Shanxi Agricultural University (39°08′20.78″ N,110° 14′18.74″ E). We utilized the drought-tolerant cultivar ‘Hequ Hongshuzi’ as experimental material [12]. Prior to sowing, seeds were surface-sterilized through a 5 min bleach treatment followed by at least three rinses with sterile distilled water. All experiments were performed in a rainout shelter to eliminate precipitation effects. The experimental design consisted of completely randomized blocks with three replicates (6 pots per replicate). Each pot was filled with 10 kg of prepared soil collected from adjacent farmland (previously uncultivated with broomcorn millet). Soil sampling was conducted using a 5 cm diameter auger to a depth of 20 cm across a 20–30 m^2^ area. The experimental farmland consisted of previously broomcorn millet-free soil, with baseline physicochemical properties detailed in Table 1. The collected soil was air-dried, homogenized, and sieved through a 2 mm mesh to remove rocks and plant residues prior to potting.

When all broomcorn millet plants reached the heading stage, we withheld water from half of the potted plants for four consecutive days, reducing soil moisture content to approximately 15% (HD). The remaining plants were maintained at 55% soil moisture (HCK). Weighing was conducted every two days to ensure soil water content. We collected rhizosphere soil samples 15 days after drought stress. Roots were rinsed with 5 mL sterile 0.9% NaCl, and the adhering soil was collected. Samples were stored at −20 °C for further analysis.

### 2.2. DNA Extraction, PCR Amplification, and Metagenomic Sequencing

We extracted genomic DNA using the NEXTFLEX^®^ Rapid DNA-Seq Kit and measured DNA purity and concentration. A paired-end (PE) library was constructed, followed by PCR amplification. Metagenomic sequencing was performed on the Illumina NovaSeq platform by Shanghai sanshu Biotechnology Co., Ltd. (Jiangsu, China)

We compared genes against multiple functional databases. Unigenes were aligned to bacterial and fungal sequences from the NCBI NR database (Version: 2021.11) using DIAMOND [38], with Blastp (*E*-value ≤ 1 × 10^−5^) [39] for homology detection. For each sequence alignment, hits with *E*-values ≤ (minimum *E*-value × 10) were retained for downstream analysis. Taxonomic annotation was performed using the LCA (Lowest Common Ancestor) algorithm implemented in MEGAN [27], yielding species abundance data across taxonomic levels. Subsequently, we conducted alpha diversity analysis, taxonomic composition profiling, PCA (Principal Component Analysis), hierarchical clustering, and LEfSe (Linear Discriminant Analysis Effect Size) to identify differentially abundant taxa between groups.

Gene classification data from each database were integrated with per-sample gene abundance tables to calculate relative abundances at different taxonomic and functional levels. The KEGG database categorizes biological information hierarchically across three levels: Level 1 (biological metabolic pathways), Level 2 (functional subcategories), and Level 3 (specific metabolic pathway maps). We generated cluster heatmaps using functional annotations and abundance data from all samples. Soil metabolites were mapped to KEGG pathways via Compound IDs, enabling visualization of metabolite-gene expression correlations in enriched pathways through heatmaps. Principal component analysis (PCA) was performed using R software.

### 2.3. Metabolite Extraction and LC-MS/MS Analysis

Soil metabolites were analyzed using an ultra-high performance liquid chromatography (UPLC) system (Vanquish, Thermo Fisher Scientific, Waltham, MA, USA) coupled with a high-resolution mass spectrometer (Q Exactive HFX, Thermo Fisher Scientific, Waltham, MA, USA). Raw data were processed with Progenesis QI software (Waters Corporation, Milford, MA, USA) for baseline filtering, peak identification, integration, retention time correction, and peak alignment. A data matrix containing retention time, mass-to-charge ratio, and peak intensity was generated and subsequently preprocessed.

In this experiment, an ultra-high performance liquid chromatography coupled with quadrupole Orbitrap mass spectrometry (UHPLC-Q Exactive HFX) was used to detect the metabolites in the samples. By matching the information such as the retention time, molecular mass (with a molecular mass error within <10 ppm) [40], and secondary fragmentation spectra of the metabolites in the local database and the commercial database, the qualitative and quantitative detection of the metabolites in the biological samples and subsequent data analysis were carried out. The entire detection process includes sample preparation, preparation of QC (quality control) samples, LC-MS/MS mass spectrometry analysis of samples, data quality control, and analysis, etc.

Raw data were processed using Progenesis QI metabolomics software (Waters Corporation, Milford, USA) for baseline filtering, peak identification, integration, retention time correction, and peak alignment, generating a data matrix of retention times, mass-to-charge ratios, and peak intensities. Metabolite identification was performed using public databases (HMDB, Metlin) and a custom-built database. The resulting data matrix was then preprocessed for subsequent statistical analysis.

### 2.4. Statistical Analysis

The experimental data were analyzed using one-way analysis of variance (ANOVA), principal component analysis, and Pearson correlation analysis with SPSS 25.0 and Microsoft Excel. Differences between treatments were assessed by the least significant difference (LSD) method at *p* < 0.05. Graphs were generated using Origin 2024.

## 3. Results

### 3.1. Microbial Community Structure

**Figure 1:**
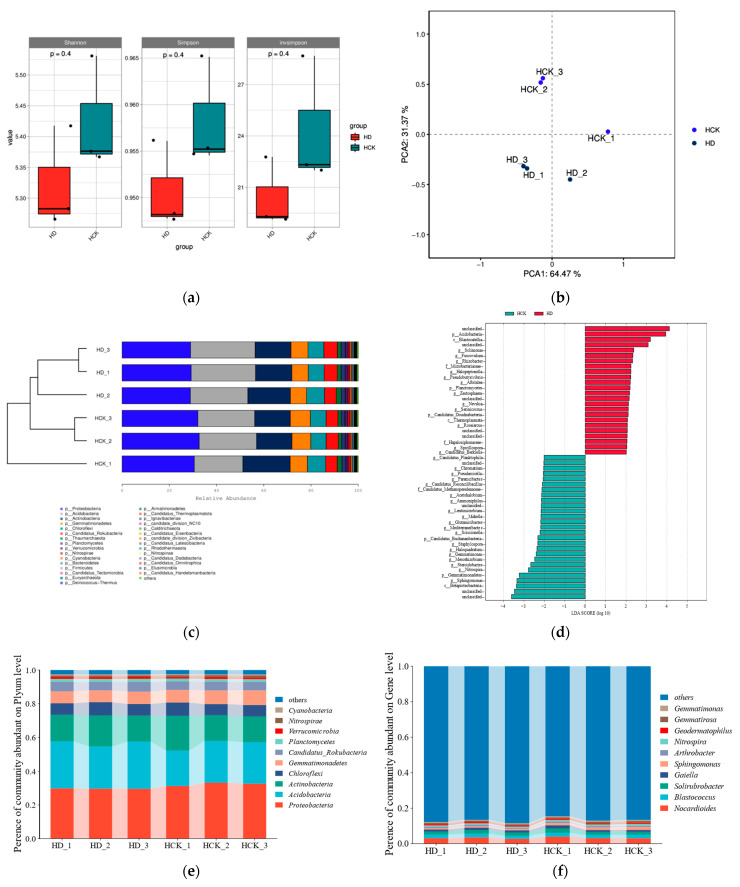

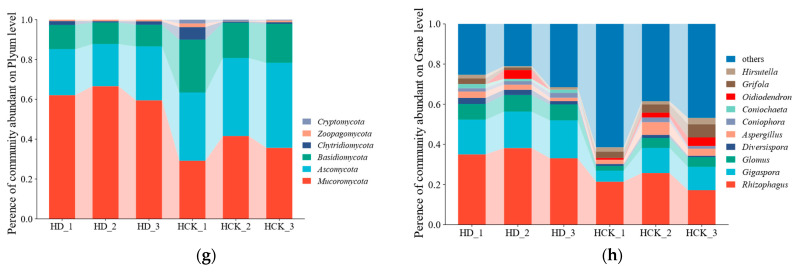
Analysis of rhizosphere microbial communities in broomcorn millet under drought stress. (**a**) Alpha and beta diversity indices; (**b**) principal component analysis (PCA) plot. (**c**) Species abundance clustering analysis. Relative abundance of rhizosphere bacteria at the (**e**) phylum and (**f**) genus levels, and fungi at the (**g**) phylum and (**h**) genus levels. (**d**) LEfSe analysis of differentially abundant taxa at the genus level between treatments. *p* < 0.05. HD: Drought stress treatment; HCK: Control treatment.

The alpha diversity analysis of the microbial community demonstrated that drought stress exerted no significant influence on the rhizosphere microbial diversity of broomcorn millet (Figure 1a). PCA and Bray–Curtis cluster analysis of the community structure showed substantial disparities in species composition between the two treatment groups (Figure 1b,c). In conclusion, drought stress modified the species composition of the microbial community without significantly influencing its diversity. A subsequent analysis of species abundance revealed a total of 175 phyla, 885 families, and 2659 genera of microorganisms.

The predominant phyla among the rhizosphere bacteria are *Proteobacteria*, *Acidobacteria*, and *Actinobacteria*, which together account for roughly 71.5% of the entire community. The top ten most abundant phyla incorporate *Chloroflexi*, *Gemmatimonadetes*, *Candidatus Rokubacteria*, *Thaumarchaeota*, *Planctomycetes*, *Verrucomicrobia*, and *Nitrospirae* (Figure 1e). Under the treatment of drought stress, the relative abundance of certain dominant bacterial species decreased slightly. Conversely, it remarkably elevated the relative abundance of *Cryptomycota*, *Acidobacteria*, *Chloroflexi*, *Candidatus Rokubacteria*, *Planctomycetes*, *Verrucomicrobia*, and *Cyanobacteria*. At the same time, it brought about a reduction in the relative abundance of *Proteobacteria*, *Actinobacteria*, *Gemmatimonadetes*, and *Nitrospirae*. At the genus level, *Nocardioides* was the most prevalent bacterium within the samples, accounting for 8.66% of the total (Figure 1f). The treatment of drought stress led to an increase in the relative abundance of the genus *Arthrobacter* and attracted microorganisms belonging to the genera *Anaerococcus*, *Candidatus*_*Hepatobacter*, *Companilactobacillus*, *Breznakibacter*, *Miniphocibacter*, *Angelakisella*, *Parendozoicomonas*, *Oribacterium*, and *Schaedlerella*. On the contrary, it caused a decrease in the relative abundance of *Nocardioides*, *Blastococcus*, *Solirubrobacter*, *Gaiella*, *Sphingomonas*, *Nitrospira*, *Geodermatophilus*, *Gemmatirosa*, and *Gemmatimonas*.

Among the rhizosphere fungi, the predominant phylum was *Mucoromycota*, accounting for 62.72% of the total (Figure 1g). The treatment of drought stress enhanced the abundance of the fungal phyla *Mucoromycota*, *Ascomycota*, and *Zoopagomycota*. Conversely, it led to a reduction in the abundance of *Basidiomycota* and *Chytridiomycota*. It is worth noting that *Cryptomycota* could not be detected under drought conditions. At the genus level, among the top 10 dominant fungal genera, only *Grifola* and *Hirsutella* exhibited a decrease in abundance. In contrast, the relative abundance of all the other genera witnessed an increase. Significantly, the genus *Coniochaeta* was detected under the condition of drought treatment (Figure 1h). Moreover, the abundances of *Rhizophagus*, *Gigaspora*, *Glomus*, and *Diversispora* demonstrated a remarkable rise in response to drought stress.

The LEfSe (Linear discriminant analysis Effect Size) analysis conducted on the two groups uncovered a total of 51 biomarker taxa at the genus level. Among them, 24 biomarkers were identified within the drought-treated group. *Solimonas*, *Fuscovulum*, *Rhizobacter*, and *Halopeptonella* stood out as the predominant biomarkers, with *Solimonas* and *Fuscovulum* demonstrating the most significant responses to drought stress (Figure 1d).

### 3.2. Functional Gene Annotation and Metabolic Pathway Analysis

**Figure 2:**
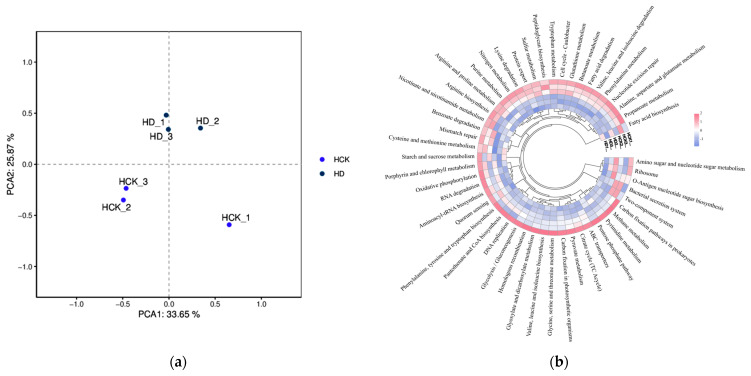
Metabolic functional analysis of the rhizosphere microorganisms of broomcorn millet. (**a**) Principal Component Analysis (PCA) plot. (**b**) Clustering heatmap. HD: Drought stress treatment; HCK: Control treatment.

The composition of microorganisms is typically intricately linked to their functional characteristics. In the present study, we carried out further annotation and classification of gene functions in both types of soil by utilizing the KEGG database. Notably, significant functional disparities were detected between the two soil groups (with PC1 accounting for 33.65% and PC2 for 25.87%), suggesting that drought stress not only brought about changes in the microbial composition of the broomcorn millet soil but also modified their metabolic functions (as depicted in Figure 2a). Subsequently, a differential analysis of functional genes was conducted for the soil samples under both experimental conditions. The results revealed that the predominant functional categories among the microbial metabolic genes affected by drought were as follows: ABC Transporters, Two-component System, Quorum Sensing, Ribosome, Oxidative Phosphorylation, Pyruvate Metabolism, Purine Metabolism, and Glyoxylate and Dicarboxylate Metabolism. Compared with the control group, drought stress significantly enhanced the carbon fixation pathways in prokaryotes as well as glycolysis/gluconeogenesis. Meanwhile, it increased the relative abundance of genes associated with ribosome biosynthesis, amino sugar and nucleotide sugar metabolism, and fructose and mannose metabolism (Figure 2b).

**Figure 3:**
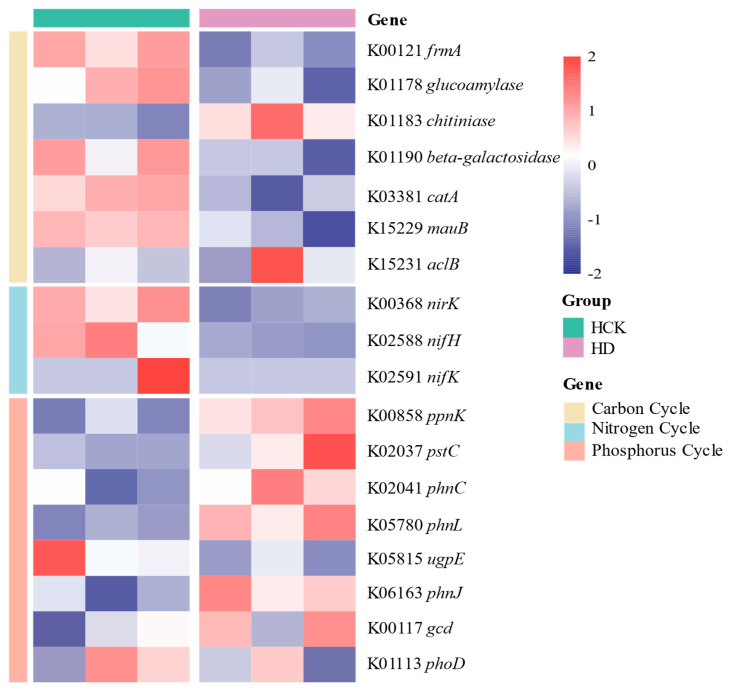
Heatmap of drought-responsive genes and functional genes.

Subsequent analysis of genes that were significantly impacted by drought stress demonstrated that drought had a remarkable inhibitory effect on the expression of genes related to carbon (C) and nitrogen (N) cycling in the soil. Significantly, the *chitinase* gene, which is involved in the chitin-degrading metabolic pathway, exhibited a significantly upregulated expression under drought conditions. Bacteria are capable of expressing this gene to secrete chitinase, which can disrupt the structures of fungal cell walls, thus offering certain protective benefits for plant growth. Moreover, the aclB gene showed a slight increase in its expression level under drought stress. This gene plays a pivotal role in microbial metabolic pathways. In contrast, the expression of most other genes was generally repressed under drought stress. Nevertheless, genes associated with phosphorus (P) cycling demonstrated heightened expression levels under drought conditions. Significantly, the gene encoding alkaline phosphatase (*phoD*) did not show any substantial changes in its expression. Microorganisms that express this gene have the ability to mobilize soil phosphorus reserves, thereby facilitating the release of bioavailable phosphorus (as illustrated in Figure 3). Genes significantly affected by drought stress may be those through which rhizospheric microorganisms resist drought and establish connections with plants via multiple pathways.

### 3.3. Composition of the Total Soil Metabolites and the Differential Metabolites in the Context of Drought Stress

**Figure 4:**
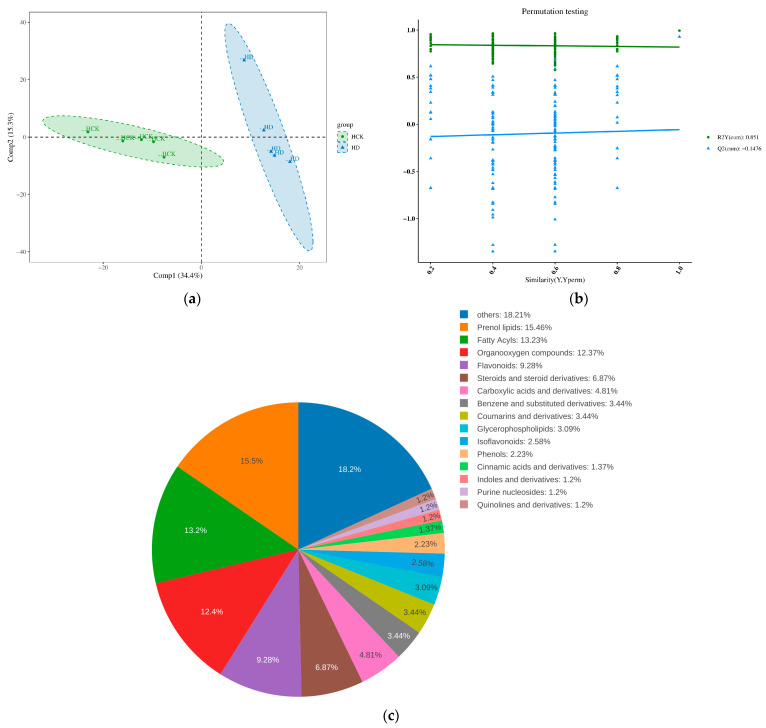
Metabolomic analysis. (**a**) PCA of metabolites from both treatment groups, (**b**) OPLS-DA score plot comparing drought-stressed and control soils. (**c**) Relative abundance of functional metabolite classes.

Principal component analysis (PCA) conducted on all the samples indicated that drought stress led to substantial changes in the metabolic composition of the rhizosphere soil of broomcorn millet (Figure 4a). To further explore and obtain more reliable insights into the drought-induced metabolic differences between the two groups, we utilized orthogonal partial least squares-discriminant analysis (OPLS-DA). The validity of the model was carefully evaluated through cross-validation, where R^2^Y represents the model’s explanatory ability for the classification variable Y, and Q reflects the predictive capacity of the model. The validation of the model resulted in an R^2^Y value of 0.28 and a corresponding Q^2^ value was < 0 (−0.1476), suggesting that the model has acceptable utility and that the data are stable and accurate (Figure 4b). Subsequent classification based on chemical taxonomy revealed that the main metabolites in the rhizosphere soil of broomcorn millet were as follows: Isoprenoid lipids accounted for 15.46%, Fatty acyls made up 13.23%, Organic oxygen compounds represented 12.37%, Flavonoids constituted 9.28%, Steroids and steroid derivatives were 6.87%, and Carboxylic acids and their derivatives were 4.81% (Figure 4c).

**Figure 5:**
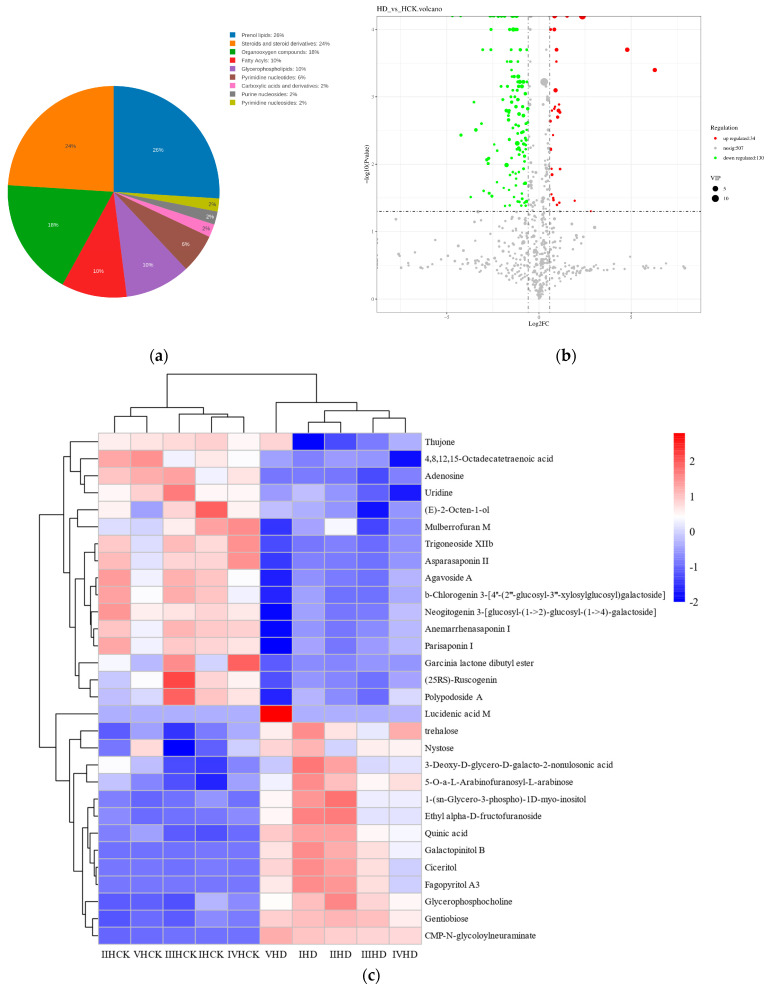
Classification and differential analysis of metabolites under drought stress. (**a**) Pie chart of differential metabolite categories: the percentages depicted represent the proportion of metabolites within each chemical class relative to the total number of identified metabolites. (**b**) Volcano plot of metabolite differences between groups: The x-axis illustrates the log_2_ (fold change), while the y-axis represents -log_10_ (*p*-value). Each dot symbolizes a metabolite, and the colors are indicative of regulation, where red denotes upregulated metabolites (“up”), green indicates downregulated metabolites (“down”), and gray signifies metabolites with no significant difference (“nosig”). The size of the dots corresponds to the VIP (Variable Importance in the Projection) value of the metabolite. (**c**) Specific metabolites and cluster analysis in drought-stressed soil: the colored blocks indicate the relative expression levels of metabolites at various positions. Specifically, red represents high expression, and blue represents low expression.

The analysis of the screened differential metabolites showed that isoprenoid lipids had the highest relative abundance (26%) in the rhizosphere soil of drought-stressed broomcorn millet (Figure 5a). They were also among the most significantly differentiated compounds. This was succeeded by steroids and steroid derivatives (24%), organic oxygen compounds (18%), fatty acyls (10%), and glycerophospholipids (10%) (Figure 5a). When compared with the control group, drought stress significantly increased the content of 34 metabolites in the broomcorn millet rhizosphere soil. These metabolites included trehalose, galactopinitol B, nystose, gentiobiose, and quinic acid. On the contrary, it was associated with a decrease in the relative abundance of 130 metabolites, such as (25RS)-ruscogenin, adenosine, and (E)-2-octen-1-ol (Figure 5b). The differential metabolites identified in the previous analysis often show biologically similar results and functions and are regulated by common metabolic pathways. Therefore, this study further carried out a cluster analysis on the differential metabolites (Figure 5c). Under drought stress, the metabolites with significantly increased levels were as follows: organic oxygen compounds: nystose, trehalose, quinic acid, 5-O-α-L-arabinofuranosyl-L-arabinose, 3-deoxy-D-glycero-D-galacto-2-nonulosonic acid, ethyl α-D-fructofuranoside, and gentiobiose. Lipids and lipid-like molecules: 4,8,12,15-octadecatetraenoic acid, 1-(sn-glycero-3-phospho)-1D-myo-inositol, ganoderic acid M, triterpenoid saponin XIIb, (25RS)-ruscogenin, polyphyllin I, neogitogenin 3-O-[glucosyl-(1→2)-glucosyl-(1→4)-galactoside], agaveside A, glycerophosphocholine, timosaponin A I, thujone, and (E)-2-octen-1-ol. Nucleosides, nucleotides, and analogs: uridine, adenosine, and CMP-N-glycolylneuraminic acid. Organic acids and derivatives: gambogic acid dilactone dibutyl ester. Other compounds: galactopinitol B, protoneodioscin II, and mulberrofuran M. This clustering emphasizes the functional groupings of metabolites that have similar responses to drought stress. Drought stress had a significant effect on the content of metabolites in the rhizospheric soil of millet, which may be influenced by both millet and microorganisms.

### 3.4. The Relationship Between Differential Metabolites and Specialized Microorganisms

**Figure 6:**
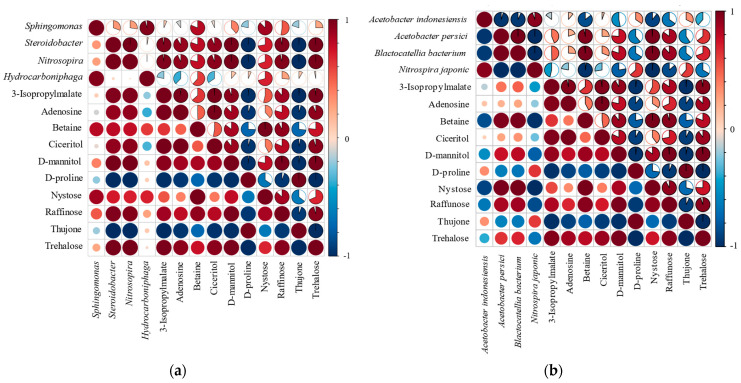

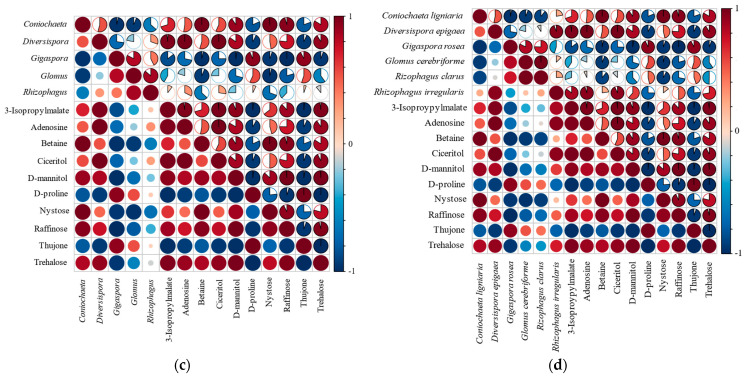
A heatmap illustrating the correlations between the soil metabolites affected by drought and the relative abundances of bacterial genera (**a**), bacterial species (**b**), fungal genera (**c**), and fungal species (**d**).

To explore the relationship between microbial taxa and soil metabolites, we performed a Pearson correlation analysis between the microbial communities and soil metabolites. The results indicated that, at the bacterial genus level: the relative abundance of *Sphingomonas* was positively correlated (*p* < 0.05) with several metabolites, namely 3-Isopropylmalate, Betaine, D-Mannitol, Nystose, Raffinose, and Trehalose. In contrast, *Sphingomonas* exhibited negative correlations (*p* < 0.05) with Adenosine, Ciceritol, D-Proline, and Thujone. These findings suggest that *Sphingomonas* may play a role in the regulation or utilization of these specific metabolites within the soil environment, potentially influencing soil metabolic processes and plant–microbe interactions. The relative abundances of *Steroidobacter* and *Nitrosospira* were positively correlated with the relative abundances of metabolites including 3-Isopropylmalate, Adenosine, Betaine, Ciceritol, D-mannitol, Nystose, Raffinose, and Trehalose. Conversely, they were negatively correlated with the relative abundances of metabolites such as D-proline and Thujone. In addition, the relative abundance of *Hydrocarboniphaga* bacteria was positively correlated with the relative abundances of metabolites like Betaine, Trehalose, D-mannitol, Nystose, and Raffinose. On the other hand, it showed a negative correlation with the relative abundances of metabolites such as 3-Isopropylmalate, Betaine, Ciceritol, D-proline, and Thujone (Figure 6a).

At the species level, the relative abundance of *Acetobacter indonesiensis* exhibited a positive correlation with the relative abundances of metabolites such as 3-Isopropylmalate, Adenosine, Ciceritol, D-proline, and Thujone. Conversely, it showed a negative correlation with the relative abundances of Betaine, D-mannitol, Nystose, Raffinose, and trehalose. In contrast, the relative abundance of *Acetobacter persici* was positively correlated with the relative abundances of metabolites including 3-Isopropylmalate, Betaine, D-mannitol, Nystose, Raffinose, and trehalose. However, it was negatively correlated with the relative abundances of Adenosine, Ciceritol, D-proline, and Thujone. The relative abundance of the *Blastocatellia* bacterium demonstrated a positive correlation with the relative abundances of metabolites like 3-Isopropylmalate, Adenosine, Betaine, Ciceritol, D-mannitol, Nystose, Raffinose, and trehalose. On the other hand, it showed a negative correlation with the relative abundances of D-proline and Thujone. In contrast, the relative abundance of *Nitrospira japonica* was positively correlated with the relative abundances of Adenosine, D-proline, and Thujone. However, it was negatively correlated with the relative abundances of 3-Isopropylmalate, Betaine, Ciceritol, D-mannitol, Nystose, Raffinose, and trehalose (Figure 6b).

At the fungal genus level, the relative abundances of *Coniochaeta* and *Diversispora* exhibit positive correlations with the relative abundances of metabolites such as 3-Isopropylmalate, Adenosine, Betaine, Ciceritol, D-mannitol, Nystose, Raffinose, and trehalose. However, they show negative correlations with the relative abundances of D-proline and Thujone. In contrast, the relative abundance of *Gigaspora* is negatively correlated with the relative abundances of 3-Isopropylmalate, Adenosine, Ciceritol, D-mannitol, Nystose, Raffinose, and trehalose. The relative abundance of *Glomus* fungi demonstrates positive correlations with the relative abundances of Adenosine, Ciceritol, D-proline, and Thujone. Nevertheless, it shows negative correlations with the relative abundances of 3-Isopropylmalate, Betaine, D-mannitol, Nystose, Raffinose, and trehalose. In contrast, the relative abundance of *Rhizophagus* fungi is positively correlated with the relative abundances of 3-Isopropylmalate, Adenosine, Ciceritol, and trehalose, yet it is negatively correlated with the relative abundances of Betaine, D-mannitol, D-proline, Nystose, Raffinose, and Thujone (Figure 6c).

At the species level, the relative abundances of *Coniochaeta ligniaria* and *Diversispora epigaea* exhibit positive correlations with the relative abundances of metabolites such as 3-Isopropylmalate, Adenosine, Betaine, Ciceritol, D-mannitol, Nystose, Raffinose, and trehalose. Meanwhile, they show negative correlations with D-proline and Thujone. Conversely, the relative abundance of *Gigaspora rosea* is positively correlated with Betaine, D-proline, Nystose, Thujone, and trehalose, but is negatively correlated with 3-Isopropylmalate, Adenosine, Ciceritol, D-mannitol, and Raffinose. At the species level, the relative abundance of *Glomus cerebriforme* shows positive correlations with the relative abundances of 3-Isopropylmalate, Adenosine, Betaine, Ciceritol, D-proline, Nystose, Thujone, and trehalose. At the same time, it exhibits negative correlations with D-mannitol, Nystose, Raffinose, and trehalose. The relative abundance of *Rhizophagus clarus* is positively correlated with 3-Isopropylmalate, Adenosine, Ciceritol, D-proline, and Thujone, yet it is negatively correlated with Betaine, D-mannitol, Nystose, Raffinose, and trehalose. As for *Rhizophagus irregularis*, its relative abundance demonstrates positive correlations with 3-Isopropylmalate, Adenosine, Ciceritol, D-mannitol, Raffinose, and trehalose, while showing negative correlations with Betaine, D-proline, Nystose, and Thujone (Figure 6d).

**Figure 7:**
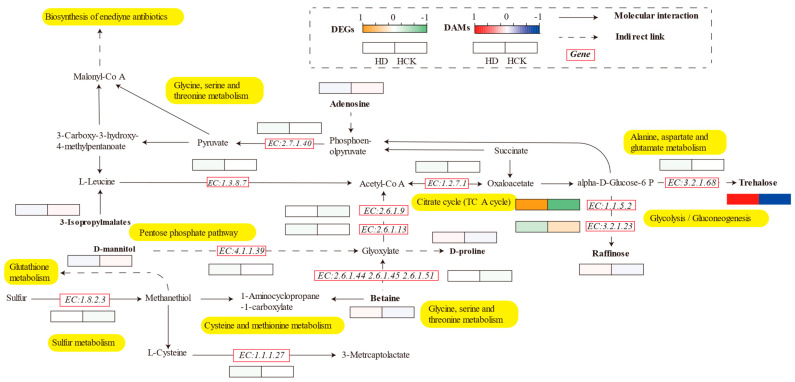
Heatmap of differentially expressed genes (DEGs) and differentially accumulated metabolites (DAMs) in enriched pathways. Each grid represents the expression levels of genes and metabolites. The orange boxes indicate upregulated genes, while the green boxes indicate downregulated genes (EC: Enzyme Commission number). The red boxes represent upregulated metabolites, and the blue boxes represent downregulated metabolites.

Compared with the control treatment, the drought treatment resulted in the upregulation of genes responsible for glyoxylate synthesis (EC: 2.6.1.44, 2.6.1.45, 2.6.1.51) in the soil, which may potentially enhance the biosynthesis of D-proline. Meanwhile, the expression of genes involved in raffinose synthesis (EC: 1.1.5.2) was increased, while the expression of the gene (EC: 3.2.1.23) was downregulated. The abundance of trehalose metabolites increased, whereas the expression of the related genes (EC: 3.2.1.68) was downregulated. The expression of genes involved in D-mannitol synthesis (EC: 4.1.1.39) decreased, which corresponded to a reduction in the relative abundance of D-mannitol, indicating that drought may have an inhibitory effect on this metabolic pathway. The genes associated with 3-isopropylmalate (EC: 1.1.1.27) and adenosine synthesis showed opposite expression patterns (Figure 7).

## 4. Discussion

Drought-tolerant crops not only undergo changes in various physiological functions [41] when resisting drought stress, but also establish interaction relationships with surrounding microorganisms to withstand drought [42]. *Azospirillum lipoferum* alleviates drought stress in maize (*Zea mays* L.) plants by producing ABA and gibberellins [43]. Water deficiency induced the interaction between microorganisms and sesame (*Sesamum indicum* L.). Treatments with arbuscular mycorrhizal fungi and bacteria enabled sesame seed yield to be comparable to that obtained under normal irrigation with N P K fertilizers [44]. Sorghum (*Sorghum bicolor* L.) shows increased yield and protein content under the action of arbuscular mycorrhizal fungi and plant growth-promoting rhizobacteria [45]. Therefore, exploring the interaction between millet, a drought-tolerant crop, and rhizospheric microorganisms is of great significance for the study of drought tolerance mechanisms.

In this study, by means of integrated metagenomic and metabolomic sequencing approaches, we aimed to explore whether broomcorn millet (*Panicum miliaceum*) and its rhizosphere microbiome could interact synergistically under drought stress to enhance their mutual drought resistance. Through the analysis of the bacterial and fungal communities in the rhizosphere of broomcorn millet under drought stress, we have observed the following: The alpha-diversity of the rhizosphere microorganisms did not show significant changes under drought stress (Figure 1a). Significant shifts in the composition of the microbial community took place (Figure 1b). The relative abundance of fungal species increased remarkably, while the abundance of bacteria remained statistically unchanged (Figure 1e–h). These results are in line with the findings reported by Cao et al. [30,35,36]. We propose that drought stress induces the selective enrichment of drought-tolerant microbial taxa, which replaces the drought-sensitive bacterial groups. There is a significant increase in the relative abundance of the fungal community [46]. Additionally, there is an environmentally mediated restructuring of the soil microbial composition. Existing studies have shown that drought stress decreases the cell density of rhizospheric bacteria while promoting the growth of fungal hyphae [47]. The relative abundances of fungi genera such as *Coniochaeta*, *Diversispora*, *Gigaspora*, *Glomus*, and *Rhizophagus* increased significantly (Figure 1g,h). Shah et al. reported that key active compounds such as D-mannitol were detected in extracts colonized by fungi of the genus *Coniochaeta*, which have potential roles in enhancing the interaction between plants and microorganisms [48]. *Diversispora spurca* can activate the antioxidant defense system of host plants to improve plant drought tolerance [49]. The tripartite interaction among fungi of the genus *Gigaspora* and their endophytic bacteria can finely regulate plant metabolism, enhance plant resilience to combined water/nutrient stress [50], and directly affect soil aggregate stability through hyphal entanglement of soil particles [51]. *Rhizophagus irregularis* can release cytokinins and auxins, and produce ethylene through the methionine and α-ketobutyrate γ-methylthio pathways [52], interacting with host plants or regulating its own development. Inoculation with *Rhizophagus intraradices* can increase the diversity of bacteria and fungi in the rhizospheric soil, and improve the composition of rhizospheric soil microbial communities in continuous cropping plants [53]. Some species of these microorganisms are AMF (arbuscular mycorrhizal fungi) [54,55,56,57], which all have a certain promoting effect on plant growth.

However, the abundances of genera such as *Sphingomonas* and *Steroidobacter* in the bacterial community significantly decreased. *Sphingomonas* sp. Hbc-6 can reshape the root bacterial community of maize under drought conditions, increasing its richness and diversity, making the rhizospheric bacterial community more complex to resist stress, and enhancing antioxidant enzyme activity and osmotic regulatory substances (proline, soluble sugars) while upregulating beneficial metabolites [58]. *Steroidobacter cummioxidans* in the genus *Steroidobacter* enriches in drought and phosphorus-free or low-phosphorus soils, enhancing the potential of phosphorus (P) cycling. This stimulation increases the abundance of microbial communities and genes involved in P cycling, thereby helping plant species in deserts to improve the utilization rate of mineral elements [59]. The decrease in the abundance of these bacteria may be influenced by drought stress. de Vries et al. confirmed that drought conditions have a far greater impact on bacterial communities than on fungal communities and may increase the diversity of fungal communities [60]. Bacterial networks in grasslands are more sensitive to drought damage [61], with stronger volatility, while fungal communities have higher resistance to changes in water availability [62] and are basically unaffected by drying. Water deficit affects the community dynamics of plant microbiomes, enhancing the competitiveness of fungi for nutrients [63]. Plant-assimilated carbon is also transferred to fungi more [64], improving microbial carbon use efficiency (CUE) [27] to maintain the growth of rhizospheric fungi. Therefore, we propose that under drought stress, beneficial fungal groups in the rhizosphere of millet are enriched to replace the bacterial groups affected by drought, so as to maintain the rhizospheric microbial community under stress. 

Element cycling promotes the symbiosis between millet and rhizospheric microorganisms under drought stress. The expression level of the *aclB* gene in carbon (C) cycling was upregulated, and genes related to phosphorus (P) cycling were significantly upregulated (Figure 3). The *aclB* gene activates ATP-citrate lyase, an enzyme encoded by this gene that catalyzes oxaloacetate and acetyl-CoA. This reaction directly connects multiple pathways of carbohydrate metabolism, links carbon, nitrogen, and energy metabolism, and plays a key role in the entire metabolic pathway of microorganisms [65]. Phosphorus (P) is an essential nutrient for all organisms, serving as a vital structural component of DNA, RNA, ATP, and phospholipids in biological membranes [66]. It plays a key role in photosynthesis, energy metabolism, regulation of various biochemical reactions, and cellular signal transduction. When phosphorus (P) precipitates in soil, it is fixed by cations, and the diffusion coefficient of phosphate ions in soil solution is low [67], making it unavailable for direct plant absorption. The expression of the alkaline phosphatase (*phoD*) gene in rhizospheric microorganisms can mobilize phosphorus in soil to release available phosphorus [59]. The *gcd* gene encodes membrane-bound quinoprotein glucose dehydrogenase (PQQGDH), which solubilizes phosphates by producing gluconate [68], thus facilitating plant uptake of P. Meanwhile, rhizospheric fungi obtain carbon produced by plant photosynthesis and provide mineral nutrients such as nitrogen and phosphorus to host plants in exchange [69], achieving plant–microbe symbiosis. In soil, phosphorus-solubilizing bacteria dissolve and convert soil phosphorus, which is then absorbed by mycorrhizal fungi and transported to host plants [70]. Shi et al. proposed that the host plant’s demand for phosphate may represent the initial motivation for AM colonization in the host plant rhizosphere [71]. AM can enhance the absorption of mineral nutrients by host plants under drought stress, including P, N, K, Zn, Cu, Mn, Fe, Ca, and Mg [72,73]. This nutrient exchange affects key soil processes and nutrient cycling, promoting the symbiosis between millet and rhizospheric microorganisms.

Gene differential expression affects related metabolic pathways and regulates the interaction between millet and rhizospheric microorganisms through the soil metabolome. The composition of metabolites such as trehalose, proline, and D-mannitol in the rhizospheric soil of millet has undergone significant changes under drought stress (Figure 3, Figure 4 and Figure 5, Q^2^ < 0). Trehalose in the glycolytic pathway enhances the tolerance of organisms to stress conditions such as drought, high salinity, and extreme temperatures [74], protecting biomolecules from denaturation [75]. It improves plant–water relations and nutrient absorption, while reducing electrolyte leakage and lipid peroxidation [76]. Exogenous application of trehalose can protect plant photosystems by regulating stomatal closure and induce the accumulation of antioxidants such as flavonoids in plants [77]. Exogenous trehalose can regulate the activities of signal pathways (cGMP-PKG, PI3K-Akt) for heterotrophic nitrification-aerobic denitrification (HN-AD) bacterial proliferation, phospholipid metabolism pathways, and aminoacyl-tRNA biosynthesis pathways. It upregulates the abundances of phosphoethanolamine (one of the glycerophospholipid metabolites), purines, and pyrimidines, stimulates bacterial aggregation and proliferation, promotes the growth of HN-AD bacteria in high-salt environments, and provides more electron donors and energy for bacteria through promoting the tricarboxylic acid (TCA) cycle, thereby enhancing the nitrogen removal performance of the system [78]. Additionally, it promotes the TCA cycle to supply electron donors and energy for bacterial carbon and nitrogen metabolism, assisting various physiological functions within organisms. Trehalose in soil can promote the expression of arbuscular mycorrhizal fungi (AMF) aquaporin (GintAQPF) and rice aquaporin (OsPIPS) to regulate water absorption in AMF and rice [79], thereby modulating the interaction between the two. Meanwhile, trehalose also increases the levels of other osmotic regulators [80]. D-mannitol, a polyol metabolite (a glycolytic bypass pathway), enables endophytic fungi to colonize orchids and promotes the microbial fructose metabolic pathway [81], thereby producing indole-3-acetic acid to facilitate host plant growth [48]. As a compatible solute, it enhances plant tolerance to salt and osmotic stress and assists plants in combating pathogens [82]. Proline, an amino acid directly linked to the tricarboxylic acid (TCA) cycle, possesses the ability to stabilize and protect biomacromolecules. It can be oxidized and coupled with the respiratory electron transport chain to generate reactive oxygen species (ROS) [83]. A reduced proline content may maintain structural integrity and function under drought stress [84]. In extreme cases, plants also store compatible solutes such as betaine and proline to maintain cellular water balance and turgor pressure [4], thus preventing water loss and resisting drought. Nader’s research has shown that drought-tolerant bacteria produce proline under drought stress [85], which helps soil absorb water through osmotic regulation and indirectly promotes water absorption and utilization by plant roots and microorganisms in the soil [86]. Proline can enhance microbial tolerance to heavy metals and improve the potential for petroleum biodegradation in the presence of heavy metals under salt stress [87]. Therefore, these substances may be the result of millet and its rhizospheric microorganisms expressing specific genes and metabolic pathways under drought stress to alter the relative abundances of trehalose, proline, and other substances, thereby enhancing their drought resistance.

**Figure 8:**
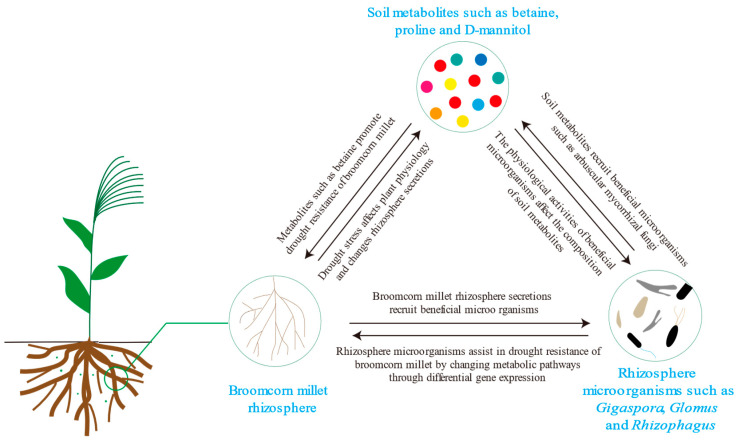
Schematic illustration of the symbiotic functional unit composed of the rhizosphere of broomcorn millet, soil metabolites, and rhizosphere microorganisms.

In this study, we developed a conceptual model depicting the interactions among broomcorn millet, soil metabolites, and rhizosphere microorganisms (Figure 8). Soil metabolites act as a crucial bridge that connects plants and microorganisms [88]. When exposed to drought stress, broomcorn millet modulates the expression of drought-resistant genes and the composition of differentially accumulated metabolites [89]. Concurrently, rhizosphere microorganisms also make synchronous adjustments to their metabolic networks [36]. Through their combined efforts, they reshape the metabolic microenvironment in the rhizosphere, establishing a positive feedback regulation loop. This two-way interaction mechanism not only brings about changes in the characteristics of the soil metabolome (for instance, significant alterations in the abundances of betaine and proline) but also has a profound impact on the host plant’s physiological response to drought stress.

## 5. Conclusions

In this study, metagenomic and metabolomic methods were utilized to explore the changes in the metabolic functions of rhizospheric microorganisms and the soil metabolites under drought stress during the flowering stage of drought-tolerant broomcorn millet. The research findings clearly illustrate that broomcorn millet is likely to affect the composition of rhizosphere microorganisms by modifying the types and abundances of soil metabolites such as D-mannitol, trehalose, and proline. Rhizospheric microorganisms may affect metabolic pathways such as glycolysis/gluconeogenesis, fructose, and mannose through differential gene expression, altering soil metabolites like trehalose and proline to assist millet in drought resistance. By doing so, they regulate soil metabolites like trehalose and proline, which in turn enhances the plant’s drought resistance. These results lay a crucial foundation for elucidating the interaction mechanisms between broomcorn millet and its rhizosphere microbiome.

## Figures and Tables

**Table 1:** Soil physicochemical properties and parameters.

Indicator	Potential of Hydrogen	Organic Matter	Total Nitrogen	Available Phosphorus	Available Potassium	Alkaline Hydrolysis Nitrogen
Parameter	8.43	8.19 g/kg	0.71 g/kg	5.89 mg/kg	97.10 mg/kg	53.59 mg/kg

## Data Availability

The data presented in this study are available on request from the corresponding author. The data are not publicly available as the project is not finished.
